# Expression of concern: Cefotaxime incorporated bimetallic silver-selenium nanoparticles: promising antimicrobial synergism, antibiofilm activity, and bacterial membrane leakage reaction mechanism

**DOI:** 10.1039/d4ra90060j

**Published:** 2024-05-28

**Authors:** Abdelrahman A. Elakraa, Salem S. Salem, Gharieb S. El-Sayyad, Mohamed S. Attia

**Affiliations:** a Botany and Microbiology Department, Faculty of Science, Al-Azhar University Nasr City Cairo 11884 Egypt salemsalahsalem@azhar.edu.eg; b Chemical Industries Department, Industrial Control Authority Cairo Egypt; c Department of Microbiology and Immunology, Faculty of Pharmacy, Galala University New Galala City Suez Egypt Gharieb.Elsayyad@gu.edu.eg Gharieb.S.Elsayyad@eaea.org.eg; d Drug Microbiology Lab., Drug Radiation Research Department, National Center for Radiation Research and Technology (NCRRT), Egyptian Atomic Energy Authority (EAEA) Cairo Egypt

## Abstract

Expression of concern for ‘Cefotaxime incorporated bimetallic silver-selenium nanoparticles: promising antimicrobial synergism, antibiofilm activity, and bacterial membrane leakage reaction mechanism’ by Abdelrahman A. Elakraa *et al.*, *RSC Adv.*, 2022, **12**, 26603–26619, https://doi.org/10.1039/D2RA04717A.

*RSC Advances* is publishing this expression of concern in order to alert readers that concerns have been raised regarding the reliability of the EDX spectra in Fig. 3 and the SEM/EDX data in Fig. 4. An investigation is underway, and an expression of concern will continue to be associated with the article until a final outcome is reached.

Laura Fisher

22nd May 2024

Executive Editor, *RSC Advances*

## Supplementary Material

